# Gastric versus postpyloric enteral nutrition in elderly patients (age ≥ 75 years) on mechanical ventilation: a single-center randomized trial

**DOI:** 10.1186/s13054-018-2092-z

**Published:** 2018-07-05

**Authors:** Youfeng Zhu, Haiyan Yin, Rui Zhang, Xiaoling Ye, Jianrui Wei

**Affiliations:** 1 Department of Intensive Care Unit, Guangzhou Red Cross Hospital, Medical College, Jinan University, Guangzhou, 510220 China; 2 Institute of Clinical Nutrition, Guangzhou Red Cross Hospital, Medical College, Jinan University, Tongfuzhong Road No. 396, Guangzhou, 510220 China

**Keywords:** Postpyloric enteral nutrition, Gastric enteral nutrition, Elderly patients, Ventilator-associated pneumonia, Mechanical ventilation

## Abstract

**Background:**

The risk of ventilator-associated pneumonia (VAP) is reduced when postpyloric enteral nutrition (EN) is administered compared to when gastric EN is administered in specific populations. In the present study, we tested the hypothesis that postpyloric EN is superior to gastric EN in reducing the incidence of VAP in elderly patients (age ≥ 75 years) who are admitted to the intensive care unit (ICU) and require mechanical ventilation.

**Methods:**

We performed a single-center randomized clinical trial involving elderly patients (age ≥ 75 years) who were admitted to the ICU and required mechanical ventilation. The patients were randomly assigned to either the postpyloric EN group or the gastric EN group. The primary outcome was the VAP rate.

**Results:**

Of the 836 patients screened, 141 patients were included in the study (70 in the postpyloric EN group and 71 in the gastric EN group). The patients in the postpyloric EN group were 82.0 (75.0–99.0) years old (male 61.4%), and those in the gastric EN group were 82.0 (75.0–92.0) years old (male 63.4%). The Acute Physiology and Chronic Health Evaluation II scores were 28.09 ± 6.75 in the postpyloric EN group and 27.80 ± 7.60 in the gastric EN group (*P* = 0.43). VAP was observed in 8 of 70 patients (11.4%) in the postpyloric EN group and in 18 of 71 patients (25.4%) in the gastric EN group, which resulted in a significant between-group difference (OR 0.38, 95% CI 0.15–0.94; *P* = 0.04). In the postpyloric EN group, there were significant reductions in vomiting (12 patients in the postpyloric EN group vs 29 patients in the gastric EN group; OR 0.30, 95% CI 0.14–0.65; *P* = 0.002) and abdominal distension (18 patients in the postpyloric EN group vs 33 patients in the gastric EN group; OR 0.40, 95% CI 0.20–0.81; *P* = 0.01). No significant differences were observed between the two groups regarding mortality and other secondary outcomes.

**Conclusions:**

Our study demonstrated that, compared with gastric EN, postpyloric EN reduced the VAP rate among elderly patients who were admitted to the ICU and required mechanical ventilation.

**Trial registration:**

Chinese Clinical Trial Registry, ChiCTR-IPR-16008485. Registered on 17 May 2016.

## Background

EN is recommended for critically ill patients [1, 2]. EN stimulates gastrointestinal blood flow, maintains the functional integrity of the gut, and prevents the translocation of bacteria [3, 4]. For critically ill patients who are unable to maintain volitional intake, it is recommended that EN should be initiated within 24–48 h after patient hemodynamics are stable [1, 2].

Ventilator-associated pneumonia (VAP), which is defined as a pneumonia occurring > 48 h after endotracheal intubation [5], is one of the most common complications that occur in critically ill patients. According to previously published studies, approximately 6–52% of patients who require mechanical ventilation are diagnosed with VAP [6–9]. All-cause mortality related to VAP ranges from 20 to 50% [5, 10]. Previous studies have demonstrated that EN may increase the risk of regurgitation and aspiration, which results in an increased risk of VAP [11, 12].

The literature is still inconclusive regarding the optimal route of EN (stomach or pylorus) to decrease the incidence of VAP. Some studies have found that postpyloric EN reduces the risk of pneumonia, including VAP, more effectively than does gastric EN [13–15]. However, in a large-scale, multicenter, randomized controlled trial (RCT) comparing gastric and postpyloric (small bowel) EN in critically ill patients, no significant differences were found in the incidences of pneumonia, nutrient delivery, or mortality between groups [16]. Additionally, a meta-analysis found no differences in clinical outcome between gastric EN and postpyloric EN (nasojejunal) among specific populations such as acute severe pancreatitis patients [17].

With the increasing age of the global population, the age of critically ill patients admitted to hospitals has also increased. In many countries, over 10% of patients in the intensive care unit (ICU) are very elderly (age ≥ 80 years) [18]. As mortality in old age has decreased, the size of the older population has grown steadily by 2% each year, which is a considerably faster rate than that of the total population [19, 20].

A previous study demonstrated that elderly patients are at a high risk of VAP [21]. However, few studies have focused on elderly patients. Although recent studies have compared gastric EN and postpyloric EN in critically ill patients, the optimal route for EN support in elderly patients (age ≥ 75 years) who are admitted to the ICU and require mechanical ventilation is unknown. In the present study, we tested the hypothesis that postpyloric EN is superior to gastric EN in reducing the incidence of VAP in elderly patients who are admitted to the ICU and require mechanical ventilation.

## Methods

### Study design and ethics

We performed a single-center, pragmatic, controlled, randomized, parallel-group clinical trial in the ICU of a tertiary university hospital (i.e., Guangzhou Red Cross Hospital of Jinan University, China). Our study received approval from the ethics committee of Guangzhou Red Cross Hospital (approval number 2014–091-01) and was conducted in accordance with the International Conference on Harmonization Good Clinical Practice Guidelines. Written informed consent was obtained from each patient or from their next of kin. The study protocol was registered with the Chinese Clinical Trial Registry (ChiCTR-IPR-16008485).

### Patients

Consecutive patients who were admitted to the ICU between April 2015 and June 2017 and were expected to require mechanical ventilation for > 48 h were evaluated for inclusion in the study. The eligibility criteria were as follows: aged ≥ 75 years, unable to undergo oral feeding, and required EN support for at least 2 days, as determined by a clinician within 24 h following ICU admission. We excluded patients who were younger than 75 years, refused to participate, had a history of gastrostomy or jejunostomy, or were unable to receive EN.

### Randomization and allocation concealment

Eligible patients were included for randomization. We used computer-generated random numbers in blocks of four to randomize patients according to the sequence of recruitment. In addition, we used sequentially numbered, opaque, sealed envelopes to maintain allocation concealment. Patients were randomly assigned in a 1:1 ratio to receive gastric EN or postpyloric EN.

Due to the nature of the present study, the double-blind method was impossible to implement. However, a staff member who was blinded to the randomization performed the statistical analyses of the outcome measures.

### Study intervention

Gastric EN and postpyloric EN tubes were placed following randomization. EN was initiated as soon as possible after randomization and with stable hemodynamic conditions and was used until the transition to exclusive oral feeding, discharge from the ICU, or death. Gastric EN tubes (Link-02-4, size CH12; Beijing L&Z Medical Technology Development Co., Ltd, Beijing, China) were administered to the patient in the ICU by either nursing staff or physicians. The position was confirmed at the bedside by air injection into the stomach or aspiration of gastric contents. If the position could not be confirmed, an abdominal X-ray scan was performed.

A commercially available, 140-cm-long, single-port tube made of radiopaque polyurethane (Corflo® nasojejunal feeding tube; Corpak Medsystems, Buffalo Grove, IL, USA) was used as the postpyloric EN tube in the present study. Similar to the insertion method described by Gerritsen et al. [22], the postpyloric EN tube was placed by physicians at the bedside with the patient in a right lateral supine position, with their head tilted at 30–45°. The path of the tip of the postpyloric EN tube was followed on a monitor screen with an electromagnetic transmitting guide wire (Cortrak® Enteral Access System; Corpak Medsystems). The postpyloric EN tube was advanced to a postpyloric position, preferably near the ligament of Treitz. Subsequently, the guide wire was removed from the postpyloric EN tube. The postpyloric EN tube was then fixed to the patient’s nose. Finally, an abdominal X-ray scan was performed to confirm the position. If the tube did not pass the pylorus after 30 min, the procedure was stopped and patient was allocated to undergo endoscopic placement.

In both groups, energy goals were set at 25 kcal per kg of ideal body weight per day, and the protein target was 1.2–2.0 g per kg of ideal body weight per day. Incidences in which the patient developed an intolerance to EN (diagnosed when vomiting, diarrhea, abdominal pain, or abdominal distension occurred) were recorded. In these cases, the rate of feeding and the amount of EN were reduced gradually as tolerated. If the nutrition goal was not reached within 7 days, parenteral nutrition was provided. Glucose control targets were set in accordance with international guidelines [1, 2, 12].

All other treatments were similar between the two groups. In addition, all other prophylactic measures for VAP, such as maintaining the head of the bed elevated at 30–45° and oral decontamination with 2% chlorhexidine solution, were provided according to international guidelines [5, 12] and at the discretion of clinicians.

### VAP diagnosis

According to the guidelines of the American Thoracic Society and the Infectious Diseases Society of America (IDSA) [5], VAP was diagnosed if there was a new lung infiltrate on chest radiograph and at least two of the following three criteria were fulfilled: body temperature > 38 °C or < 36 °C, peripheral white blood cell count >10,000/μl or < 4000/μl, and purulent sputum. Otherwise, after 48 h of ventilation for those patients with pneumonia at admission, VAP was also diagnosed if the aforementioned situation emerged or there were new microbial culture results from distal respiratory specimens that were different from those at admission, and colonization was excluded.

The timeframe for the diagnosis of VAP was after 48 h of mechanical ventilation. Furthermore, new-onset pneumonia within 48 h after extubation was also considered to be VAP [5].

During the period of hospitalization, VAP was independently diagnosed by two senior physicians who treated these patients. To reduce the possibility of subjective researcher bias, these physicians were not involved in data collection. Disagreements were resolved by consulting a third senior physician in our center. After completion of the study, all cases that were diagnosed with VAP were rechecked by another senior respiratory disease physician who made a final decision and did not know the allocation of the groups.

### Data collection

Once patients were enrolled, demographic data were collected by independent staff members who were not involved in the trial. Physicians and nursing staff who treated these patients were not involved in data collection. The following clinical parameters were recorded after enrollment: age, gender, primary diagnosis, need for inotropic therapy, need for renal replacement therapy, Sequential Organ Failure Assessment (SOFA) score, severity of illness as assessed by the Acute Physiology and Chronic Health Evaluation II (APACHE II) score, and nutrition risk evaluation as assessed by the Nutritional Risk Score 2002 (NRS 2002).

Adverse events due to tube insertion were also recorded, and included perforation, insertion into the trachea, cardiac arrest, need for sedatives during the procedure, tachycardia, dyspnea, and nasal mucosal bleeding. Furthermore, we defined perforation, insertion into the trachea, and cardiac arrest as serious adverse events.

### Endpoints

The primary outcome of the study was the rate of VAP for the duration of mechanical ventilation. The secondary outcomes included the incidence of vomiting, diarrhea, abdominal pain, abdominal distension, achievement of the energy goal by EN during the first 7 days of ICU admission, duration of mechanical ventilation, length of ICU and hospital stay, and mortality in the ICU and hospital.

### Statistical analysis

The sample size calculation was based on previously published studies in which the incidences of VAP were approximately 6–52% [6–9]. The incidence of VAP in our ICU in 2014 was approximately 38%; therefore, we assumed a VAP incidence of 38% in the gastric EN group, and an enrollment of 70 patients in each group would achieve an 80% statistical power and a 5% level of significance (one-sided) to detect a VAP reduction of 50 percentage points in the postpyloric EN group.

We analyzed all data in the study according to the intention-to-treat principle. Categorical variables of demographic data, clinical outcomes, or other laboratory parameters are presented as frequencies, and continuous variables are presented as the mean ± standard deviation (SD) or median with range of minimum and maximum values. We used the Pearson chi-square test or Fisher’s exact test to analyze categorical variables as appropriate. Continuous outcomes were analyzed using analysis of variance for normally distributed data or nonparametric tests for non-normally distributed data. We also conducted post-hoc subgroup analyses for several baseline variables such as age, gender, SOFA score, APACHE II score, NRS 2002, and need for a vasopressor. Odds ratios (ORs) with 95% confidence intervals (CIs) are reported for all categorical outcomes. We also performed a post-hoc multivariate logistic regression to analyze the risk factors for VAP, which are presented as ORs and 95% CIs. We included covariates in the multivariate logistic regression model if the associated *P* value was < 0.15 in the univariate model, if the variables were previously considered to be potential confounders, or if the variables could complicate the relationships of outcomes in biology. The variables tested in the regressions were age, gender, APACHE II score, SOFA score, NRS 2002, vomiting, diarrhea, abdominal pain, abdominal distension, achievement of the energy goal by EN during the first 7 days of ICU admission, duration of mechanical ventilation, and length of ICU and hospital stay. After the univariate analyses, a forward (LR), step-wise removal process was applied. *P* < 0.05 was considered to indicate statistical significance. The statistical analyses were conducted using SPSS 13.0 (IBM Corp., Armonk, NY, USA).

## Results

**Fig. 1:**
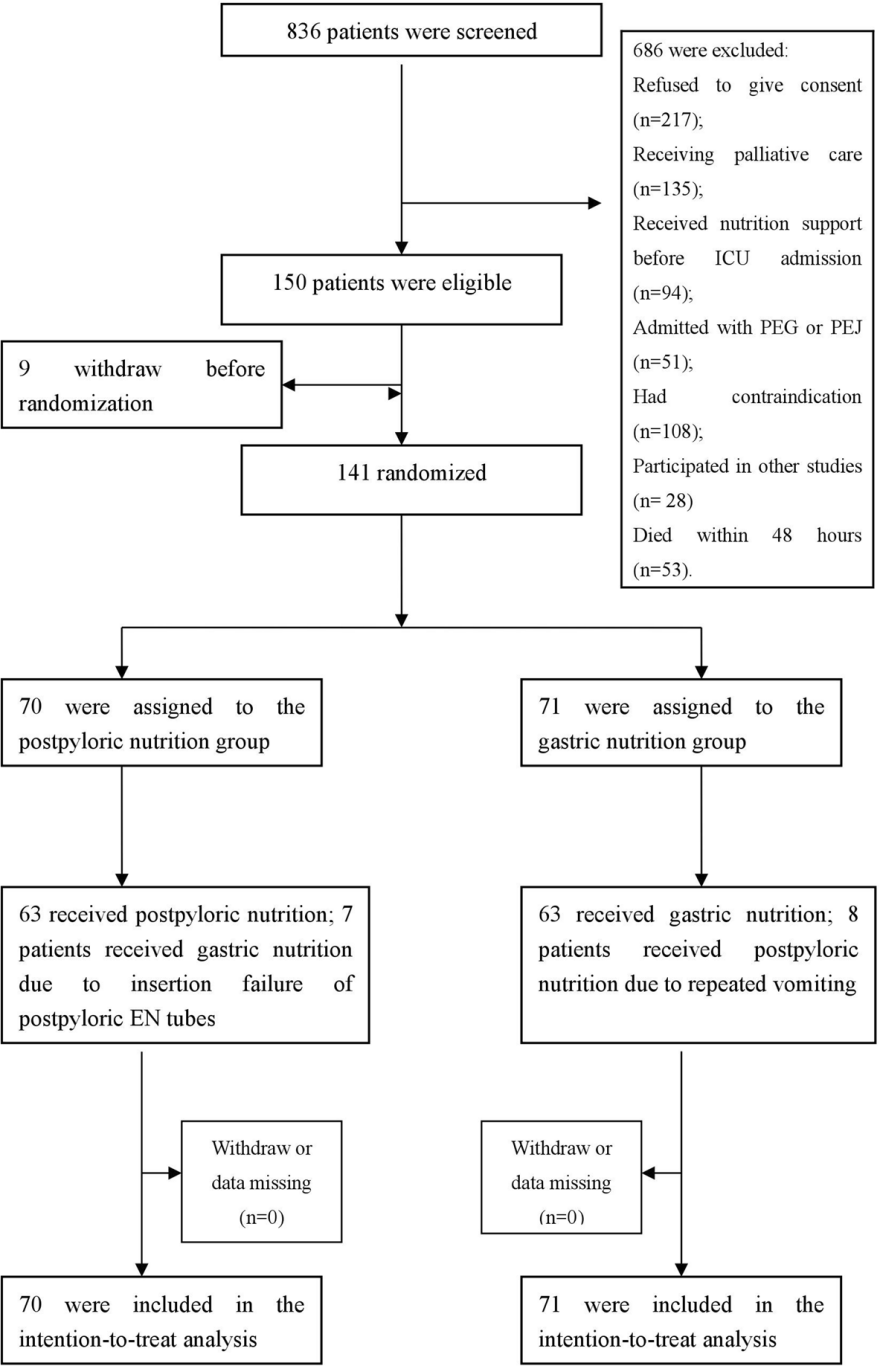
Study screening, selection, and randomization process. ICU intensive care unit, PEG percutaneous endoscopic gastrostomy, PEJ percutaneous endoscopic jejunostomy

From April 1, 2015 to June 5, 2017, 836 patients admitted to the ICU of Guangzhou Red Cross Hospital were screened for eligibility. Of these patients, 150 were eligible, including nine patients who subsequently withdrew before randomization. This resulted in an intention-to-treat population of 141 patients (70 in the postpyloric EN group and 71 in the gastric EN group). The screening and selection process is shown in Fig. 1. In the postpyloric EN group, postpyloric feeding tubes were successfully placed in 63 of 70 patients (90%). In the remaining seven patients, due to the failure of bedside placement and intolerance of endoscopic placement, nasogastric tubes were inserted instead. In the gastric EN group, eight patients with initially placed nasogastric tubes were changed to postpyloric feeding tubes due to repeated vomiting. The demographic data and clinical characteristics of the patients in the two study groups were similar (Table 1).

**Table 1:** Demographic data and clinical characteristics of patients at baseline

Characteristic	Postpyloric EN group (*n* = 70)	Gastric EN group (*n* = 71)	*P* value
Age (years), median (range)	82.0 (75.0–99.0)	82.0 (75.0–92.0)	0.99
≥ 80 years, *n* (%)	48/70 (68.6)	50/71 (70.4)	0.81
Male, *n* (%)	43 (61.4)	45 (63.4)	0.81
NRS 2002,^a^ median (range)	5.0 (4.0–7.0)	5.0 (4.0–7.0)	0.81
High risk of malnutrition, *n*/total *n* (%)	62/70 (88.6)	61/71 (85.9)	0.64
APACHE II score,^b^ mean ± SD	28.09 ± 6.75	27.80 ± 7.60	0.43
SOFA score,^c^ median (range)	8.0 (3.0–16.0)	8.0 (4.0–17.0)	0.28
Need for vasopressor, *n* (%)	35 (50.0)	31 (43.7)	0.45
Comorbidities, *n*/total *n* (%)			
Pneumonia	55/70 (78.6)	52/71 (73.2)	0.46
COPD	13/70 (18.6)	10/71 (14.1)	0.47
CHD	26/70 (37.1)	36/71 (50.7)	0.11
Congestive heart failure	5/70 (7.14)	7/71 (9.86)	0.56
Diabetes	12/70 (17.1)	11/71 (15.5)	0.79
Stroke	22/70 (31.4)	19/71 (26.8)	0.54
Number of comorbidities, *n*/total *n* (%)			
2	15/70 (21.4)	14/71 (19.7)	0.80
3	24/70 (34.3)	30/71 (42.3)	0.33
≥ 4	29/70 (41.4)	24/71 (33.8)	0.35

*APACHE II* Acute Physiology and Chronic Health Evaluation II, *CHD* coronary heart disease, *COPD* chronic obstructive pulmonary disease, *EN* enteral nutrition, *NRS 2002* Nutritional Risk Score 2002, *SD* standard deviation, *SOFA* Sequential Organ Failure Assessment

^a^Total score, based on age, severity of illness, and nutrition state, ranges from 0 to 7, with higher scores indicating a greater degree of malnutrition. We defined high risk of malnutrition as NRS 2002 score ≥ 5

^b^Acute Physiology Score, based on data regarding physiological function obtained during the first 24 h after admission to the ICU, ranges from 0 to 60, with higher scores indicating greater severity of illness. The total score, which is based on acute physiology, age, and severe coexisting illnesses, ranges from 0 to 71, with higher scores indicating greater severity of illness

^c^Scores range from 0 to 24, with higher scores indicating a greater degree of organ failure. SOFA score calculated using data obtained within 24 h before randomization

The patients were 82.0 (75.0–99.0) years old in the postpyloric EN group (male 61.4%) and 82.0 (75.0–92.0) years old in the gastric EN group (male 63.4%). Over two-thirds of these patients were very elderly (≥ 80 years): 68.6% (48 patients) in the postpyloric EN group vs 70.4% (50 patients) in the gastric EN group (*P* = 0.81). Pneumonia was the most common disease in these patients, followed by coronary heart disease, stroke, and chronic obstructive pulmonary disease. Most patients in both groups had more than two comorbidities (97.1% (68 patients) in the postpyloric EN group vs 95.8% (68 patients) in the gastric EN group; *P* = 1.00), and over one-third of the patients had at least four comorbidities (41.4% (29 patients) in the postpyloric EN group vs 33.8% (24 patients) in the gastric EN group; *P* = 0.35). The illness severity of these patients was very high (APACHE II score 28.09 ± 6.75 in the postpyloric EN group vs 27.80 ± 7.60 in the gastric EN group; *P* = 0.43). Most of the patients had a high risk of malnutrition (defined as NRS 2002 ≥ 5).

**Fig. 2:**
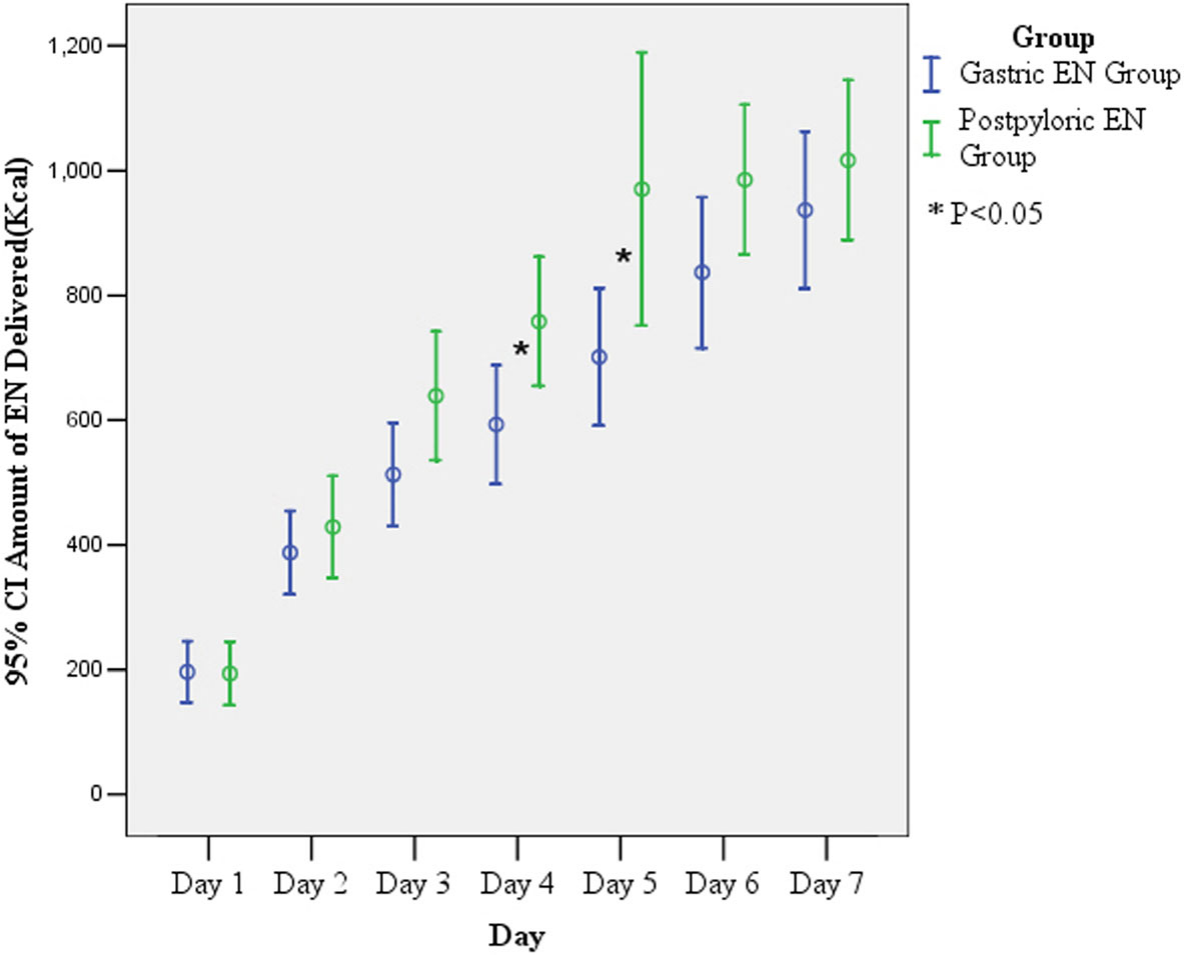
Amount of EN delivered each study day over the first 7 days in each group. CI confidence interval, EN enteral nutrition

The times from randomization to the initiation of EN were 24.2 ± 10.1 h in the postpyloric EN group and 24.4 ± 10.6 h in the gastric EN group (*P* = 0.46). The amounts of EN delivered each study day in the first 7 days in each group are presented in Fig. 2.

### Primary outcome

Overall, 8 of 70 patients (11.4%) in the postpyloric EN group and 18 of 71 patients (25.4%) in the gastric EN group had VAP, which resulted in a significant between-group difference (OR 0.38, 95% CI 0.15–0.94; *P* = 0.04) (Table 2). In terms of ventilation days, the VAP rates were 8.45 of 1000 ventilation days in the postpyloric EN group and 17.79 of 1000 ventilation days in the gastric EN group (*p* = 0.049).

**Table 2:** Primary and secondary outcomes

Outcome	Postpyloric EN group (*n* = 70)	Gastric EN group (*n* = 71)	OR (95% CI)	*P* value
Primary outcome				
VAP, *n*/total *n* (%)	8/70 (11.4)	18/71 (25.4)	0.38 (0.15–0.94)	0.04
Secondary outcomes				
Vomiting, *n*/total *n* (%)	12/70 (17.1)	29/71 (41.4)	0.30 (0.14–0.65)	0.002
Abdominal distension, *n*/total *n* (%)	18/70 (25.7)	33/71 (46.5)	0.40 (0.20–0.81)	0.01
Diarrhea, *n*/total *n* (%)	6/70 (8.57)	4/71 (5.63)	1.57 (0.42–5.82)	0.50
Abdominal pain, *n*/total *n* (%)	4/70 (5.71)	3/71 (4.22)	1.37 (0.30–6.38)	0.69
Achievement of energy goal by EN in the first 7 days, *n*/total *n* (%)	40/70 (57.1)	32/71 (45.1)	1.63 (0.84–3.16)	0.15
Need for renal replacement therapy, *n*/total *n* (%)	13/70 (18.6)	19/71 (26.8)	0.62 (0.28–1.39)	0.25
Mortality in ICU, *n*/total *n* (%)	32/70 (45.7)	40/71 (56.3)	0.65 (0.34–1.27)	0.21
Mortality in hospital, *n*/total *n* (%)	37/70 (52.9)	43/71 (60.6)	0.73 (0.37–1.42)	0.36
Duration of mechanical ventilation (h), median (range)	239.5 (58–1087)	244.0 (51–2170)	0.47	
Length of ICU stay (h), median (range)	306.5 (67–1265)	368.0 (60–2228)	0.55	
Length of hospital stay (days), median (range)	19.0 (3–53)	16.0 (3–125)	0.57	
Time from randomization to EN start (h), mean ± SD	25.86 ± 23.59	22.80 ± 21.24	0.11	

OR derived from multivariate logistic regression analysis

*CI* confidence interval, *EN* enteral nutrition, *ICU* intensive care unit, *OR* odds ratio, *SD* standard deviation, *VAP* ventilator-associated pneumonia

### Secondary outcomes

The postpyloric EN group exhibited significantly less vomiting (17.1% (12 patients) in the postpyloric EN group vs 41.4% (29 patients) in the gastric EN group; OR 0.30, 95% CI 0.14–0.65; *P* = 0.002) and abdominal distension (25.7% (18 patients) in the postpyloric EN group vs 46.5% (33 patients) in the gastric EN group; OR 0.40, 95% CI 0.20–0.81; *P* = 0.01) than the patients in the gastric EN group. However, no significant differences were observed between the two groups for the other parameters, including abdominal pain, diarrhea, achievement of the energy goal by EN during the first 7 days, need for renal replacement therapy, duration of mechanical ventilation, length of ICU and hospital stay, and mortality in the ICU or hospital (Table 2).

### Post-hoc analysis

**Fig. 3:**
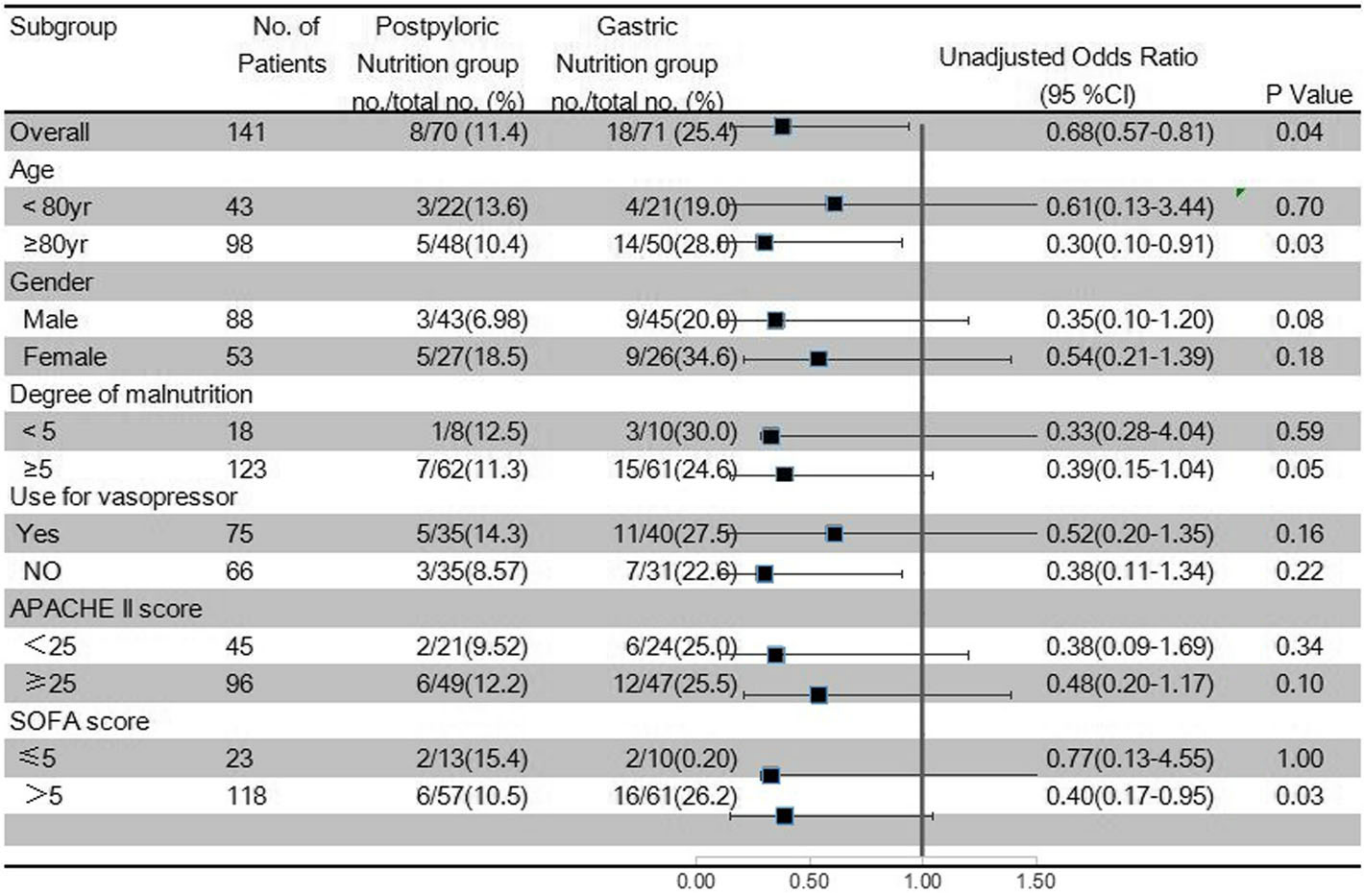
Subgroup analyses with respect to VAP. Degree of malnutrition quantified according to Nutritional Risk Score 2002. APACHE II Acute Physiology and Chronic Health Evaluation II, CI confidence interval, SOFA Sequential Organ Failure Assessment

Subgroup analyses with respect to VAP according to age, gender, SOFA score, APACHE II score, NRS 2002, and need for a vasopressor revealed that the patients in the postpyloric EN group with a SOFA score > 5 or aged ≥ 80 years may have benefited more than the other subgroups (Fig. 3).

The post-hoc multivariate logistic regression found that vomiting, abdominal distension, and the duration of mechanical ventilation were risk factors for VAP and that the achievement of the energy goal during the first 7 days was a protective factor against VAP (Table 3).

**Table 3:** Multivariate logistic regression model for risk factors of VAP

Variable	Odds ratio (95% CI)	*P* value
Vomiting	6.77 (2.09–22.00)	0.001
Abdominal distension	11.00 (3.09–39.19)	0.000
Duration of mechanical ventilation	1.002 (1.00–1.004)	0.018
Achievement of energy goal in first 7 days	0.29 (0.09–0.93)	0.038

OR derived from multivariate logistic regression analysis

*CI* confidence interval, *OR* odds ratio, *VAP* ventilator-associated pneumonia

### Adverse events

No patients reported serious adverse events, including perforation, insertion into the trachea, and cardiac arrest, due to the insertion of the tube (Table 4). In the postpyloric EN group, four patients exhibited tachycardia, and three patients exhibited dyspnea during the endoscopic placement procedure due to the failure of bedside placement; these issues were all resolved via the placement of nasogastric tubes instead. The other minor adverse events were similar between the two groups and were quickly resolved.

**Table 4:** Adverse events between postpyloric and gastric EN groups

Variable	Postpyloric EN group (*n* = 70)	Gastric EN group (*n* = 71)	*P* value
Perforation, *n* (%)	0 (0)	0 (0)	
Insertion into trachea, *n* (%)	0 (0)	0 (0)	
Cardiac arrest, *n* (%)	0 (0)	0 (0)	
Need for sedatives during procedure, *n* (%)	15 (21.4)	8 (11.3)	0.10
Tachycardia,^a^ *n* (%)	4 (5.7)	2 (2.8)	0.44
Dyspnea,^a^ *n* (%)	3 (4.3)	1 (1.4)	0.37
Nasal mucosal bleeding, *n* (%)	4 (5.7)	3 (4.2)	0.72

*EN* enteral nutrition

^a^Defined as that increased over the range of 15%

## Discussion

Our study demonstrated that among elderly patients on mechanical ventilation, the postpyloric EN route resulted in a reduced VAP rate compared with that observed for the gastric EN route (8 of 70 patients (11.4%) in the postpyloric EN group vs 18 of 71 patients (25.4%) in the gastric EN group; OR 0.38, 95% CI 0.15–0.94; *P* = 0.04). Additionally, the patients who received postpyloric EN exhibited significant reductions in vomiting (12 of 70 patients (17.1%) in the postpyloric EN group vs 29 of 71 patients (40.8%) in the gastric EN group; OR 0.30, 95% CI 0.14–0.65; *P* = 0.002) and abdominal distension (18 of 70 patients (25.7%) in the postpyloric EN group vs 33 of 71 patients (46.5%) in the gastric EN group; OR 0.40, 95% CI 0.20–0.81; *P* = 0.01) compared with the patients who received gastric EN. However, no significant differences were observed between the two groups in the other parameters such as diarrhea, abdominal pain, use of renal replacement support, duration of mechanical ventilation, length of ICU or hospital stay, or ICU or hospital mortality. Only approximately 50% of patients who received EN reached their energy targets during the first 7 days, and no significant difference was found between the two groups (40 of 70 patients (57.1%) in the postpyloric EN group vs 32 of 71 patients (45.1%) in the gastric EN group; OR 1.63, 95% CI 0.84–3.16; *P* = 0.15).

Postpyloric EN has been demonstrated to offer benefits in specific populations. Acosta-Escribano et al. [13] evaluated the efficacies of postpyloric EN and gastric EN in mitigating the incidence of VAP in severe traumatic brain injury (TBI) patients. Their study demonstrated that postpyloric EN reduces the incidence of VAP and decreases the incidence of gastric residuals. Additionally, a meta-analysis involving five randomized controlled studies revealed that severe TBI patients who were administered postpyloric EN exhibited a reduced incidence of VAP (RR 0.52, 95% CI 0.34–0.81; *P* = 0.003; *I*^2^ = 0.0%) and fewer total complications compared with patients who were administered gastric EN [23].

However, the effect of postpyloric EN on the VAP rate has not been assessed in elderly patients who are admitted to the ICU and require mechanical ventilation. In the present study, our results demonstrated the benefits of postpyloric EN in reducing VAP in this population. These benefits may be due to the decreased occurrences of vomiting and abdominal distension associated with postpyloric EN. A previous study showed that gastroparesis was common in critically ill patients, resulting in feeding intolerance. Approximately 50% of these critically ill patients experienced delayed gastric emptying, which placed the patients at a higher risk of aspiration if gastric feeding was provided [24]. Aspiration is the primary cause of VAP. Risk factors for aspiration include age ≥ 70 years, gastroesophageal reflux, and mechanical ventilation, all of which are common in elderly patients. Therefore, elderly patients who require mechanical ventilation represent a population at high risk for VAP [21, 25]. Our multivariate logistic regression analysis also confirmed that vomiting and abdominal distension were risk factors for VAP.

In the present study, the VAP rate was somewhat different from those reported in previous studies. Wang et al. [26] reported that 10.4–16.0% of pneumonia patients who required mechanical ventilation were diagnosed with VAP during 2005–2011 in a randomly selected national sample. Mathai et al. [27] reported that 38% of patients develop VAP infections and that the VAP incidence is 40.1/1000 mechanical ventilation days in an adult ICU of a tertiary care hospital in northern India. Various factors could explain these differences. First, the population included in our study was elderly, which differed from previous studies. Second, in the present study, the included patients had more risk factors for VAP, such as high APACHE II scores, chronic obstructive pulmonary disease (COPD), acute renal replacement therapy, and sepsis [5, 28].

Although a lower VAP rate would be expected to shorten the duration of mechanical ventilation or the length of ICU or hospital stay, our study showed no differences with regard to these parameters. This finding may be due to the increased severity of illness (APACHE II score 28.09 ± 6.75 in the postpyloric EN group vs 27.80 ± 7.60 in the gastric EN group; Table 1) and number of comorbidities in the study population. Our results were similar to those of a previous meta-analysis reported in the 2016 American Society for Parenteral and Enteral Nutrition guidelines [1]. Furthermore, the mortality of very elderly patients was high. Garrouste-Orgeas et al. [29] reported that the ICU and hospital mortalities of very elderly patients (≥ 80 years) were 50% and 62.5%, respectively. Our study showed similar results. The high mortality rate may offset the benefits of postpyloric EN with respect to duration of mechanical ventilation or length of ICU or hospital stay. However, some factors not included in the present study, such as frailty, may influence comparisons between these two groups. A recent study demonstrated that frailty was present in 43.1% of very elderly ICU patients (age ≥ 80 years) and was an independent risk factor for 30-day mortality (32.6%) [18]. However, the sample size was not calculated to include these secondary outcomes, which may limit the ability to evaluate the effects of these results.

The strengths of the present study were that it was rigorously conducted and that the baseline measurements were well balanced between groups. However, there were some limitations of our study. First, this was a single-center study, and the sample size was relatively small. Therefore, the effects of postpyloric EN could be overestimated. However, our results were similar to previous studies with respect to VAP and mortality rates [19, 30]. This demonstrates that the results of our study were reliable. Furthermore, because few studies to date have focused on VAP in elderly patients, the present study can be a useful reference for preventing VAP in this population and provide a basis for further studies of this topic that will be conducted due to the increasing population of elderly patients. Second, the diagnosis of VAP in the present study primarily relied on clinical criteria and did not use biomarkers such as serum PCT. This may influence the accuracy of VAP diagnosis. However, according to the 2016 IDSA guidelines for HAP and VAP, the false-negative rates of using serum PCT plus clinical criteria for the diagnosis of VAP were up to 33%, and the false-positive rate was 17% [5]. Therefore, biomarkers plus clinical criteria are not recommended by the new guidelines for the diagnosis of VAP. Third, patients with pneumonia at admission were not excluded in the present study. A great number of patients in both groups suffered pneumonia at admission (78.6% in the postpyloric EN group and 73.2% in the gastric EN group; Table 1). This condition may interfere with the VAP diagnosis. However, in clinical practice, respiratory disease is one of the main reasons for needing mechanical ventilation. In a previous study by Chastre et al. [21], respiratory disease was found to be an independent predictor of VAP (RR 2.8, 95% CI 1.1–7.5). In other randomized controlled studies related to VAP therapy or prevention, patients with an a-priori diagnosis of respiratory disease (including pneumonia) were also involved, and these patients accounted for a high proportion of the mechanical ventilation patients [31, 32]. We thought that if we excluded patients who had pneumonia at admission, the validity of the study would be decreased. Therefore, these patients were not excluded from our study. To reduce the influence of previous pneumonia on VAP diagnosis, after the completion of the study, all cases who were diagnosed with VAP were rechecked by another senior respiratory disease physician who made a final decision and did not know the allocation of the groups. Fourth, it is difficult to successfully place postpyloric EN tubes in a patient. A recently published prospective study demonstrated that the success rate of the blind bedside placement of postpyloric EN tubes is no more than 50% [33]. Moreover, in the present study, seven patients in the postpyloric EN group received nasogastric tubes due to insertion failure, which decreased the adherence to the study protocol and may have influenced our results. Hu et al. [34] reported that the oral administration of metoclopramide or domperidone could improve the success rate of postpyloric EN tube placement in critically ill patients. Recently, Lv et al. [35] reported a new rescue method for the blind bedside placement of postpyloric tubes following initial placement failure in critically ill patients that involves the use of intravenous metoclopramide. This method increased the success rate of placement and should be considered in further studies.

## Conclusion

Our study demonstrated that, compared with gastric EN, postpyloric EN reduced the VAP rate in elderly patients (age ≥ 75 years) who were admitted to the ICU and required mechanical ventilation. Further studies are needed to confirm these results.

## Abbreviations

APACHE II: Acute Physiology and Chronic Health Evaluation II
COPD: Chronic obstructive pulmonary disease
EN: Enteral nutrition
NRS 2002: Nutritional Risk Score 2002
OR: Odds ratio
RCT: Randomized controlled trial
SOFA: Sequential Organ Failure Assessment
VAP: Ventilator-associated pneumonia

## References

[1] Taylor BE, McClave SA, Martindale RG, Warren MM, Johnson DR, Braunschweig C (2016). Guidelines for the provision and assessment of nutrition support therapy in the adult critically ill patient: Society of Critical Care Medicine (SCCM) and American Society for Parenteral and Enteral Nutrition (A.S.P.E.N.). Crit Care Med.

[2] McClave SA, DiBaise JK, Mullin GE, Martindale RG (2016). ACG clinical guideline: nutrition therapy in the adult hospitalized patient. Am J Gastroenterol.

[3] Doig GS, Heighes PT, Simpson F (2009). Early enteral nutrition, provided within 24 h of injury or intensive care unit admission, significantly reduces mortality in critically ill patients: a meta-analysis of randomised controlled trials. Intensive Care Med.

[4] Jabbar A, Chang WK, Dryden GW (2003). Gut immunology and the differential response to feeding and starvation. Nutr Clin Pract.

[5] Kalil AC, Metersky ML, Klompas M, Muscedere J, Sweeney DA (2016). Management of adults with hospital-acquired and ventilator-associated pneumonia: 2016 clinical practice guidelines by the Infectious Diseases Society of America and the American Thoracic Society. Clin Infect Dis.

[6] Kohlenberg A, Schwab F, Behnke M, Geffers C, Gastmeier P (2010). Pneumonia associated with invasive and noninvasive ventilation: an analysis of the German nosocomial infection surveillance system database. Intensive Care Med.

[7] Joseph NM, Sistla S, Dutta TK, Badhe AS, Parija SC (2009). Ventilator-associated pneumonia in a tertiary care hospital in India: incidence and risk factors. J infect Dev Ctries.

[8] Xie DS, Xiong W, Lai RP, Liu L, Gan XM, Wang XH (2011). Ventilator-associated pneumonia in intensive care units in Hubei Province, China: a multicentreprospective cohort survey. J Hosp Infect.

[9] Shorr AF, Chan CM, Zilberberg MD (2011). Diagnostics and epidemiology in ventilator associated pneumonia. Ther Adv Respir Dis.

[10] Melsen WG, Rovers MM, Groenwold RH (2013). Attributable mortality of ventilator-associated pneumonia: a meta-analysis of individual patient data from randomised prevention studies. Lancet Infect Dis.

[11] Drakulovic MB, Torres A, Bauer TT, Nicolas JM, Nogue S, Ferrer N (1999). Supine body position as a risk factor for nosocomial pneumonia in mechanically ventilated patients: a randomised trial. Lancet.

[12] Rhodes A, Evans LE, Alhazzani W, Levy MM, Antonelli M, Ferrer R (2017). Surviving Sepsis Campaign: International Guidelines for Management of Sepsis and Septic Shock: 2016. Intensive Care Med.

[13] Acosta-Escribano J, Fernández-Vivas M, Grau Carmona T (2010). Gastric versus transpyloric feeding in severe traumatic brain injury: a prospective, randomized trial. Intensive Care Med.

[14] Kearns PJ, Chin D, Mueller L (2000). The incidence of ventilator-associated pneumonia and success in nutrient delivery with gastric versus small intestinal feeding: a randomized clinical trial. Crit Care Med.

[15] Hsu CW, Sun SF, Lin SL (2009). Duodenal versus gastric feeding in medical intensive care unit patients: a prospective, randomized, clinical study. Crit Care Med.

[16] Davies AR, Morrison SS, Bailey MJ (2012). ENTERIC study investigators; ANZICS clinical trials group. A multicenter, randomized controlled trial comparing early nasojejunal with nasogastric nutrition in critical illness. Crit Care Med.

[17] Zhu Y, Yin H, Zhang R, Ye X, Wei J (2016). Nasogastric nutrition versus nasojejunal nutrition in patients with severe acute pancreatitis: a meta-analysis of randomized controlled trials. Gastroenterol Res Pract.

[18] Flaatten H, De Lange DW, Morandi A, Andersen FH, Artigas A, Bertolini G (2017). The impact of frailty on ICU and 30-day mortality and the level of care in very elderly patients (≥ 80 years). Intensive Care Med.

[19] Boumendil A, Guidet B (2006). Elderly patients and intensive care medicine. Intensive Care Med.

[20] Chin-Yee N, D’Egidio G, Thavorn K, Heyland D, Kyeremanteng K (2017). Cost analysis of the very elderly admitted to intensive care units. Crit Care.

[21] Chastre J, Fagon JY (2002). Ventilator-associated pneumonia. Am J Respir Crit Care Med.

[22] Gerritsen A, de Rooij T, Dijkgraaf MG, Busch OR, Bergman JJ, Ubbink DT (2015). Electromagnetic guided bedside or endoscopic placement of nasoenteral feeding tubes in surgical patients (CORE trial): study protocol for a randomized controlled trial. Trials.

[23] Wang D, Zheng SQ, Chen XC, Jiang SW, Chen HB (2015). Comparisons between small intestinal and gastric feeding in severe traumatic brain injury: a systematic review and meta-analysis of randomized controlled trials. J Neurosurg.

[24] Fruhwald S, Kainz J (2010). Effect of ICU interventions on gastrointestinal motility. Curr Opin Crit Care.

[25] McClave SA, DeMeo MT, DeLegge MH (2002). North American summit on aspiration in the critically ill patient: consensus statement. J Parenter Enter Nutr.

[26] Wang Y, Eldridge N, Metersky ML, Verzier NR, Meehan TP, Pandolfi MM (2014). National trends in patient safety for four common conditions, 2005-2011. N Engl J Med.

[27] Mathai AS, Phillips A, Isaac R (2016). Ventilator-associated pneumonia: a persistent healthcare problem in Indian intensive care units!. Lung India.

[28] Kasuya Y, Hargett JL, Lenhardt R, Heine MF, Doufas AG, Remmel KS (2011). Ventilator-associated pneumonia in critically ill stroke patients: frequency, risk factors, and outcomes. J Crit Care.

[29] Garrouste-Orgeas M, Timsit JF, Montuclard L, Colvez A, Gattolliat O, Philippart F, Rigal G, Misset B, Carlet J (2006). Decision-making process, outcome, and 1-year quality of life of octogenarians referred for intensive care unit admission. Intensive Care Med.

[30] Lee SH, Lee TW, Sunmi J, Yoo J-W, Lee SJ, Ji Cho Y (2017). Outcomes of very elderly (≥80 years) critical-ill patients in a medical intensive care unit of a tertiary hospital in Korea. Korean J Intern Med.

[31] Damas P, Frippiat F, Ancion A, Canivet JL, Lambermont B, Layios N (2015). Prevention of ventilator-associated pneumonia and ventilator-associated conditions: a randomized controlled trial with subglottic secretion suctioning. Crit Care Med.

[32] Reignier J, Mercier E, Le Gouge A, Boulain T, Desachy A, Bellec F (2013). Effect of not monitoring residual gastric volume on risk of ventilator-associated pneumonia inadults receiving mechanical ventilation and early enteral feeding: a randomized controlled trial. JAMA.

[33] Chen W, Sun C, Wei R, Zhang Y, Ye H, Chi R (2018). Establishing decision trees for predicting successful postpyloric nasoenteric tube placement in critically ill patients. JPEN.

[34] Hu B, Ye H, Sun C, Zhang Y, Lao Z, Wu F (2015). Metoclopramide or domperidone improves post-pyloric placement of spiral nasojejunal tubes in critically ill patients: a prospective, multicenter, open-label, randomized, controlled clinical trial. Crit Care.

[35] Lv B, Hu L, Chen L, Hu B, Zhang Y, Ye H (2017). Blind bedside postpyloric placement of spiral tube as rescue therapy in critically ill patients: a prospective, tricentric, observational study. Crit Care.

